# Supporting medication discontinuation: provider preferences for interventions to facilitate deprescribing

**DOI:** 10.1186/s12913-017-2391-0

**Published:** 2017-06-28

**Authors:** Amy Linsky, Mark Meterko, Kelly Stolzmann, Steven R. Simon

**Affiliations:** 1 Section of General Internal Medicine (152G), VA Boston Healthcare System, 150 S. Huntington Ave, Boston, MA 02130 USA; 2 Center for Healthcare Organization and Implementation Research, VA Boston Healthcare System and ENRM Veterans Affairs Medical Center, 150 S. Huntington Ave, Boston, MA USA; 3 Section of General Internal Medicine, Boston Medical Center, Boston, MA USA; 4 Performance Measurement, VHA, Office of Reporting, Analytics, Performance, Improvement and Deployment (RAPID), Bedford, MA USA; 5 Health Law, Policy and Management, Boston University School of Public Health, Boston, MA USA

**Keywords:** Ambulatory care, Medical decision making, Medical safety, Physician decision support

## Abstract

**Background:**

One approach to prevent adverse drug events is to discontinue (“deprescribe”) medications that are outdated, not indicated, or of limited benefit relative to risk for a particular patient. However, there is little guidance to clinicians about how to integrate the process of deprescribing into the workflow of clinical practice. We sought to determine clinical prescribers’ preferences for interventions that would improve their ability to appropriately and proactively discontinue medications.

**Methods:**

We conducted a national web-based survey of 2475 prescribers [physicians, nurse practitioners (NP), physician assistants (PA), and clinical pharmacy specialists] practicing in US Veterans Affairs (VA) primary care clinics. One survey question presented 15 potential changes to medication-related practices and respondents ranked their top three choices for changes that would “most improve [their] ability to discontinue medications.” We summed the weighted rankings for each of the 15 response options. Preferences were determined for the whole sample and within subgroups of respondents defined by demographic and background characteristics, medication-relevant experience, and beliefs.

**Results:**

Among the 326 respondents who provided rankings, the top choice for a change that would help improve their ability to discontinue medications was “Requiring all medication prescriptions to have an associated ‘indication for use.’” This preference was followed by “Assistance with follow-up of patients as they taper or discontinue medications is performed by another member of the Patient Aligned Care Team (PACT)” and “Increased patient involvement in prescribing decisions.” This combination of options, albeit in varying rank order, was the most commonly selected, with 250 respondents (77%) who answered the question including at least one of these items in their three highest ranked choices, regardless of their demographics, experience, or beliefs.

**Conclusions:**

Continued efforts to improve clinicians’ ability to make prescribing decisions, especially around deprescribing, have many potential benefits, including decreased pharmaceutical and health care costs, fewer adverse drug events and complications, and improved patient involvement and satisfaction with their care. Future work, whether as research or quality improvement, should incorporate clinicians’ preferences for interventions, as greater buy-in from front-line staff leads to better adoption of changes.

## Background

Adverse drug events (ADEs) are associated with increased healthcare utilization, costs, and morbidity [1, 2]. One approach to prevent ADEs is to discontinue medications that are outdated, not indicated, or of limited benefit relative to risk for a particular patient [3]. This activity, also known as deprescribing, has been defined as a “systematic process of identifying and discontinuing drugs in instances in which existing or potential harms outweigh existing or potential benefits within the context of an individual patient’s care goals, current level of functioning, life expectancy, values, and preferences” [4].

While discontinuing a medication can be considered “doing less,” it often requires more provider effort than simply continuing the status quo. Literature supports the clinical feasibility and safety of medication discontinuation but does not necessarily provide information about how to identify which medications can be deprescribed or how to proceed in typical practice [5]. One example is the “Good Palliative-Geriatric Practice” framework developed by Garfinkel et al., but this effort was a feasibility study and the method appears to be labor intensive, making its routine use difficult [6]. Others have described the success and failures of prescribing interventions but give little direct guidance to clinicians; [7, 8] one exception has been developed in New Zealand [9].

Qualitative research exploring prescribers’ understanding and approach toward discontinuation revealed concerns about the negative effects of inappropriate medication use and overall support for the idea of discontinuing unnecessary medications [10]. However, clinicians also discussed the many factors that impede their ability to deprescribe, including patient complexity, clinical uncertainty, and shared management with other healthcare providers, all of which can contribute to “clinical inertia” around medication discontinuation [10, 11].

The development of clinical interventions that are integrated into the clinical workflow may facilitate appropriate deprescribing decisions. However, to increase the likelihood of success of any such intervention, the interest and support of the end-users (in this case, the prescribing clinicians) need to be garnered. To that end, we undertook the present study to survey a national sample of primary care providers and clinical pharmacy specialists in the US Department of Veterans Affairs (VA) to determine preferences for interventions that would improve their ability to discontinue medications.

## Methods

### Instrument development

**Fig. 1:**
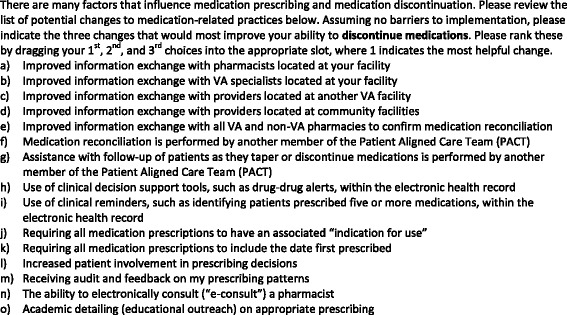
Survey Question and Proposed Intervention Options Note: The presentation order of response items (a) through (o) was randomized to minimize bias associated with selecting options appearing earlier in a list

We developed the Provider Perceptions of Medication Discontinuation survey instrument according to accepted standards of survey design [12]. Full details of instrument development have been described elsewhere [13]. Briefly, we generated a pool of 75 items based on a conceptual model of 10 dimensions related to medication discontinuation we previously developed using qualitative methods and augmented with literature findings [10]. These items were evaluated in a modified Delphi process by a seven-member panel of researchers and primary care providers (PCP), including experts in survey development and medication safety, to create a draft survey. We then conducted cognitive interviews with physicians, nurse practitioners, and clinical pharmacy specialists, including in-depth probes to understand how respondents interpret the items and response options [14]. To assess providers’ general attitudes regarding the use of medication, we included the previously validated Beliefs about Medications Questionnaire Overuse scale [15]. Providers were also asked to indicate their current overall comfort level with deciding to discontinue a medication on a 0–10 scale ranging from “not at all comfortable” to “completely comfortable.” The survey included 56 items related to medication discontinuation, plus 8 demographic items. One of the discontinuation-related questions presented respondents with 15 potential changes to medication-related practices and asked them to rank their top three choices for the changes that would “most improve [their] ability to discontinue medications.” These change options were developed from qualitative interviews with clinicians, literature review, and expert opinion. The full list of options can be seen in Fig. 1. The survey continued with an optional open-ended invitation to write in other suggestions for interventions.

### Pilot study and psychometric evaluation

#### Sample

We surveyed VA clinical providers with prescribing privileges. Our sampling frame was the Primary Care Management Module, a centralized VA database which contains clinical information for all PCPs. From this listing we identified all providers nation-wide with the title of Physician-Primary Care, Physician-Attending, Primary Care Provider, Nurse Practitioner (NP), or Physician Assistant (PA). Using another centralized database, we identified Clinical Pharmacy Specialists by selecting “Pharmacy Service Providers” who had Primary Care clinical encounters. Based on the sample size required to assess the psychometric properties of the survey, we randomly selected 2500 providers from a total of more than 9951 eligible individuals, oversampling NP/PAs and pharmacists to ensure adequate representation and to enable comparisons across the three provider types (MD/DO, NP/PA, PharmD) and stratifying evenly across four geographic regions (East, South, Midwest, West).

#### Survey administration

We sent each provider an email introducing the survey objectives and containing a link to the survey website. If an email was undeliverable, we selected a replacement participant of the same provider type and geographic stratum. Non-respondents received up to two reminder emails at one-week intervals. All responses were anonymous. The presentation order of the 15 options for potential changes to medication-related practice was randomized among respondents to minimize bias toward the options near the top of the list.

#### Analysis strategy

We first examined the distributions of respondents’ demographics and beliefs to identify subgroups with sufficiently robust representation. For some variables with multiple response options we collapsed categories to facilitate data analysis by creating relatively equal-sized subgroups. Based on these results, we dichotomized the following variables for use in subsequent analyses: gender (male vs. female), race (non-Hispanic white vs. other), clinical effort (1–7 vs. 8–10 half-day clinic sessions per week), prior non-VA experience (yes vs. no), beliefs about medication overuse (agree/strongly agree vs. neutral/disagree/strongly disagree), and perceptions of patients’ ability to manage their own health (more vs. less ability). Similarly, we trichotomized the following variables: age (<50 years vs. 50–59 years vs. ≥60 years), provider type (MD/DO vs. NP/PA vs. PharmD), self-rated comfort with discontinuation (≤6 vs. 7–8 vs. ≥9), experience within VA (≤4 years vs. 5–9 years vs. ≥10 years), frequency of experiencing uncertainty about the indication for a patient’s medication (never/rarely vs. sometimes vs. often/usually), experience with discontinuing medications initiated by other providers (never/rarely vs. sometimes vs. often/usually), and experience with patient activation (low vs. medium vs. high).

Because the responses to the main outcome (potential intervention approaches) were ranked, we assigned a weight of 3 for any option that was selected as a first choice, a weight of 2 for any option selected as a second choice, and a weight of 1 for any option that was selected as a third choice. We then summed the weighted rankings for each of the fifteen response options. Preferences were determined for the sample as a whole and by categories of respondents’ demographics and beliefs as described above. Write-in responses were reviewed for suggestions that differed from the options included in the survey instrument.

All analyses were performed using SAS software, Version 9.3 (SAS Institute, Inc). This study protocol was approved by the Institutional Review Board of the VA Boston Healthcare System.

## Results

A total of 411 clinical providers completed the online questionnaire. After accounting for unreachable individuals (*n* = 25), the response rate was 17% (411/2475). Non-responders were more likely than responders to be physicians, as compared with NP/PAs or pharmacists, but were otherwise similar with respect to age, gender, and geographic region. Details regarding respondent demographics are in Table 1. Regarding data quality, the median percent of missing responses per item on the substantive questions was 11.7 (range 0.01–16%); the median percent missing on the demographic questions was 17.2 (range 16–19%).

**Table 1:** Self-Reported Respondent Demographics

Characteristic	n^a^ (%)
Age, years	
≤ 49	128 (37)
50–59	125 (37)
≥ 60	89 (26)
Gender	
Male	162 (48)
Female	174 (52)
Race	
Non-Hispanic White	243 (72)
Other	93 (28)
Provider Type	
Physician	304 (74)
Nurse Practitioner or Physician Assistant	68 (17)
Clinical Pharmacy Specialist	39 (9)
Clinic sessions per week	
< 8	159 (52)
≥ 8	175 (48)
Number of years working in VA	
≤ 4	99 (29)
5–9	84 (25)
≥ 10	158 (46)
Prior experience working in non-VA setting	268 (79)

^a^Respondent counts do not sum to 411 due to missing responses.

With respect to medication discontinuation, clinicians rated themselves on the higher end of the comfort scale, with 73% scoring 7 or higher out of 10. A total of 11% agreed or strongly agreed that medications are overused, and 27% rarely or never encountered uncertainty about the indication for a medication. Additional details about respondents’ beliefs can be seen in Table 2.

**Table 2:** Respondent Beliefs and Perceptions

Factor	n^a^ (%)^b^
Self-rated comfort with medication discontinuation (0–10 scale)	
≤ 6	92 (27)
7–8	138 (40)
≥ 9	116 (34)
Beliefs about medication overuse	
Agree/Strongly Agree	44 (11)
Neutral/Disagree/Strongly Disagree	355 (89)
Perceptions of patients’ ability to manage their own health	
More Ability	119 (33)
Less Ability	246 (67)
Frequency of experiencing uncertainty about the indication for a patient’s medication	
Never/rarely	107 (27)
Sometimes	209 (53)
Often/usually	80 (20)
Experience with discontinuation of medications initiated by other providers	
Never/rarely	51 (14)
Sometimes	295 (83)
Often/usually	10 (3)
Experience with patient activation	
Low	144 (36)
Medium	134 (33)
High	126 (31)

^a^Respondent counts do not sum to 411 due to missing responses.

^b^Percentages may not sum to 100 due to rounding.

Among the 326 respondents who provided rankings, the top choice for a change that would help improve their ability to discontinue medications was “Requiring all medication prescriptions to have an associated ‘indication for use.’” This preference was followed by “Assistance with follow-up of patients as they taper or discontinue medications is performed by another member of the Patient Aligned Care Team (PACT)” and “Increased patient involvement in prescribing decisions” (Table 3). This combination of options, albeit in varying rank order, was the most commonly selected, regardless of prescriber demographics, experience, or beliefs; overall, 250 (77%) of respondents who provided rankings included at least one of these in their top three highest ranked choices.

**Table 3:** Preferences for Interventions to Improve Ability to Deprescribe Medications

	First Choice	Second Choice	Third Choice
All Respondents			
All (326)	Indication for use	Assistance with follow-up	Patient involvement
Age			
<49 years (118)	Indication for use	Assistance with follow-up	Information exchange with all pharmacies
50–59 years (120)	Indication for use	Assistance with follow-up	Patient involvement
≥60 years (83)	Indication for use	Patient involvement	Assistance with follow-up
Gender			
Male (151)	Indication for use	Assistance with follow-up	Patient involvement
Female (165)	Assistance with follow-up	Indication for use	Patient involvement
Race			
White (235)	Assistance with follow-up	Indication for use	Patient involvement
Non-White (81)	Indication for use	Patient involvement	Clinical decision support
Provider type			
Physician (243)	Indication for use	Assistance with follow-up	Patient involvement
Nurse Practitioner or Physician Assistant (53)	Indication for use	Assistance with follow-up	Patient involvement
Clinical Pharmacy Specialist (30)	Indication for use	Information exchange with all pharmacies	Improved information exchange with providers located at community facilities
Number of clinic sessions per week			
≤7 (148)	Assistance with follow-up	Indication for use	Patient involvement
8–10 (167)	Indication for use	Patient involvement	Assistance with follow-up
Number of years working in VA			
≤4 (94)	Indication for use	Information exchange with all pharmacies	Assistance with follow-up
5–9 (80)	Assistance with follow-up	Indication for use	Patient involvement
≥10 (146)	Indication for use	Assistance with follow-up	Patient involvement
Prior experience working in a non-VA setting			
Yes (250)	Indication for use	Assistance with follow-up	Patient involvement
No (69)	Assistance with follow-up	Patient involvement	Indication for use
Self-rated comfort with medication discontinuation (0–10 scale)			
Low, 0–6 (83)	Assistance with follow-up	Indication for use	Information exchange with all pharmacies
Medium, 7–8 (126)	Assistance with follow-up	Indication for use	Patient involvement
High 9–10 (108)	Indication for use	Assistance with follow-up	Information exchange with all pharmacies
Beliefs about medication overuse			
Neutral/disagree/strongly disagree (288)	Indication for use	Assistance with follow-up	Patient involvement
Agree/strongly agree (38)	Patient involvement	Indication for use	Information exchange with all pharmacies
Perceptions of patients’ ability to manage their own health			
Less (223)	Indication for use	Assistance with follow-up	Patient involvement
More (101)	Indication for use	Patient involvement	Assistance with follow-up
Frequency of experiencing uncertainty about the indication for a patient’s medication			
Never/Rarely (83)	Indication for use	Assistance with follow-up	Information exchange with all pharmacies
Sometimes (173)	Assistance with follow-up	Indication for use	Patient involvement
Often/Usually (68)	Indication for use	Patient involvement	Assistance with follow-up
Experience with discontinuing medications initiated by other providers			
Never/rarely (46)	Patient involvement	Indication for use	Clinical decision support
Sometimes (271)	Indication for use	Assistance with follow-up	Patient involvement
Often/usually (8)	Assistance with follow-up	Indication for use	Patient involvement
Experience with patient activation			
Low (117)	Indication for use	Assistance with follow-up	Information exchange with all pharmacies
Medium (108)	Assistance with follow-up	Indication for use	Patient involvement
High (97)	Patient involvement	Indication for use	Assistance with follow-up

Indication for use = Requiring all medication prescriptions to have an associated “indication for use”

Assistance with follow-up = Assistance with follow-up of patients as they taper of discontinue medications is performed by another member of the Patient Aligned Care Team (PACT)

Patient involvement = Increased Patient involvement in prescribing decisions

Information exchange with all pharmacies = Improved information exchange with all VA and non-VA pharmacies to confirm medication reconciliation

Clinical decision support = Use of clinical decision support tools, such as drug-drug alerts, within the electronic health record

There were a few exceptions to this combination of preferred interventions. “Improved information exchange with all VA and non-VA pharmacies to confirm medication reconciliation” was selected more often than “increased patient involvement” among respondents who were younger (age ≤ 49 years), had worked in the VA for ≤4 years, had never/rarely encountered indication uncertainty, had low *or* high self-rated comfort, or had lower experience with patient activation. “Improved information exchange” was also chosen by respondents who believed medications were overused, rather than selecting “assistance with follow-up of patients.” Providers who reported rarely discontinuing medications by other clinicians or who were non-white preferred “Use of clinical decision support tools, such as drug-drug alerts, within the electronic health record” instead of “assistance with follow-up.” Finally, clinical pharmacy specialists ranked having an “indication for use” first, followed by “improved information exchange,” but then ranked “Improved information exchange with providers located at community facilities” third.

Fifty-one respondents (14%) wrote additional suggestions or comments. The majority were variations on the 15 proposed interventions, expressing support for division of labor among PACT members, improving medication reconciliation, and the benefit of additional time with patients. Others requested explicit clinical guidelines and criteria for deprescribing.

## Discussion

We surveyed a national sample of physicians, nurse practitioners, physician assistants and clinical pharmacists in VA to assess their preferences for interventions to support deprescribing. Including the indication for use on all prescriptions was the highest ranked choice to support clinical providers’ efforts to discontinue medications, suggesting that this intervention may have wide acceptance among many clinicians across VA. Other highly rated interventions included increased teamwork to monitor patients after deprescribing and including patients in the decision-making process.

Much as clinical uncertainty can impede a physician from intensifying medication, it can also hinder discontinuation. If a patient is tolerating a medication and the indication (clinical reason for a medication’s use) is unclear, there may be reluctance to stopping it without a compelling reason. In a study by Straand et al. looking at patient-physician agreement about the decision and rationale for discontinuing a medication, up to 30% of the medicines had an unclear indication [16]. One factor contributing to this uncertainty could be the frequent use of off-label prescribing in practice, which one study found accounts for over 20% of all prescription medications [17]. Certain medications, such as gabapentin and amitriptyline (an anticonvulsant and antidepressant, respectively) are used off-label up to 80% of the time [17]. Given that one of the first steps in the deprescribing process is to review the rationale for use so as to identify potentially inappropriate medications, not knowing the indication for a medication is an early obstacle to discontinuation [3, 8].

Many patients have medications prescribed by multiple providers in separate healthcare systems and filled in different pharmacies [18]. This system-level complexity also contributes to loss of information about indication for use [19]. In the Straand study, over half of the medicines discontinued were prescribed by someone other than the discontinuing provider [16]. Compounding the impact of such information loss is potential reluctance among many clinicians to stop a medicine initiated by another provider [3, 19]. Knowing the indication for a prescription may provide reassurance to the discontinuing provider that deprescribing is appropriate.

Others have proposed similar interventions, such as including a “review by” date or “planned duration of use” that would prompt review of a medication’s continued appropriateness [4]. Likewise, the Centers for Medicare & Medicaid Services requires nursing home patients to have an indication for all medications [3]. One intervention study prompted inpatient providers to document the indication for three specific medications if an appropriate one was not found on the problem list [20]. This study found including an associated indication at the time of prescribing increased, albeit these were often for off-label or non-evidence based use. Evaluation of this type of intervention in the outpatient setting and determining its effect on future discontinuation is still needed.

Including the indication with prescriptions has known and potential benefits in addition to aiding deprescribing decisions by the clinician. It may allow for therapeutic substitutions, in that the clinician can evaluate if there are new treatment options that were unavailable when the medication was initiated. In situations where prescriptions are handwritten, including a diagnosis allows a pharmacist to confirm that the medication is the one intended, especially when the handwriting is poor [21]. It may also help with drug utilization reviews [22]. Related to the findings in our study, adding an indication may increase patient knowledge [23]. One of the top ranked interventions was to increase patient involvement in decision-making; educating patients at the *initiation* of a medication should facilitate their participation in subsequent deprescribing discussions. In a review of 12 intervention studies by Ostini et al., including the patient was a key component of successful deprescribing [24]. While it is unclear whether the indication should be written in medical or layman’s terms, either approach could potentially educate the patient. Including the indication on the printed prescription and/or the label of the medication packaging might allow for more feedback from the patient about whether the clinical problem still exists or whether the medication ever alleviated symptoms. It has been suggested that conversations about deprescribing may also lead to increased discussion about overall goals of care; [25] given the high cost of medical care at end of life, providing the indication and consequent deprescribing may have even greater implications on healthcare utilization.

Another highly preferred intervention was involvement of other members of the Patient Aligned Care Team, VA’s patient centered medical home. This finding speaks to the time constraints felt by many PCPs and their desire for workload support [19]. In a corollary to the increased monitoring of patients often required when initiating or increasing the dose of a medication, discontinuing or decreasing the dose may lead to similar increased requirements for surveillance, communication and office visits. Knowing that this follow-up could be managed by another team member (e.g., a nurse) may make prescribing clinicians more amenable to enacting medication changes. Indirectly, redistributing tasks may free up clinicians to give more time to re-evaluating the continued need for a medication rather than just renewing a prescription. In one study, the majority of outpatient prescriptions were renewals (72%) as compared to initiation of a new medication [26]. It may also create more time in the clinical visit to have the conversation with the patient about discontinuing a medication, a process which might, in fact, be less time consuming if the patient is more knowledgeable.

These findings are of use to clinical practice managers in helping to guide future changes to clinical practice. The consistency of the preferences among the providers in our study, regardless of provider characteristics, experience or beliefs, indicates that these intervention options are more likely to be accepted by a wider range of providers. Further, including an indication could be integrated into electronic health records and computerized order entry systems. Other health information technologies and clinical decision support systems have been shown to reduce the initiation of inappropriate prescribing [27, 28]. Requiring the prescriber to select from approved indications and commonly used off-label reasons, with an option to write in alternate rationale, could lead to widespread inclusion of indication for use.

Several limitations to our study should be noted. The survey response rate was low, which could reduce the ability to generalize to all VA clinicians. Multiple factors may have contributed to this, including clinicians’ busy schedules and the ability to only access the survey from behind the institutional firewall. Clinicians have lower response rates than the general population, [29, 30] and there was no incentive provided. However, other than having a higher proportion of physicians, the non-responders were similar to the responders with regard to age, sex and geographic region. Also, the survey was conducted in VA and may not be generalizable to other health care settings. Future studies outside VA should examine prescribers’ preferences for interventions to support deprescribing.

## Conclusions

When asked to indicate their preferences for the changes in clinical practice that would best support their efforts to identify and discontinue unnecessary mediations, primary care providers and clinical pharmacy specialists most strongly endorsed a requirement that all medications have an associated indication for use. Other highly preferred interventions were division of labor among care team members and including the patient in medical decision making. Future work, whether as research or quality improvement, should incorporate these preferences, as greater buy-in from front line staff leads to better adoption of changes [31]. While the exact design and implementation of such interventions is yet to be determined, continued efforts to improve the ability of PCPs to make medication decisions, especially around deprescribing, have many potential benefits, including decreased pharmaceutical and health care costs, fewer adverse drug events and complications, and improved patient involvement and satisfaction with their care.

## Abbreviations

ADE: Adverse drug events
NP: Nurse Practitioner
PA: Physician Assistant
PACT: Patient Aligned Care Team
PCP: Primary Care Provider
VA: Veterans Affairs
